# Supplementary material for: Magnaporthe oryzae CK2 Accumulates in Nuclei, Nucleoli, at Septal Pores and Forms a Large Ring Structure in Appressoria, and Is Involved in Rice Blast Pathogenesis
Source: Front Cell Infect Microbiol. 2019 Apr 17;9:113. doi: 10.3389/fcimb.2019.00113 (PMC6478894; doi:10.3389/fcimb.2019.00113)
Supplement: Table S1 — Primers used in this study. [file Table_1.docx]

Table S1 Primers used in this study

| Primer name | The sequence of primer（5’ 3’） |
| --- | --- |
| 3696qRTF | CGTCAACTACCAGAAATGCG |
| 3696qRTR | TGACGGAGTCTTGCTCTGTG |
| 446qRTF | GCAGAGGTGTCGGAGGAAT |
| 446qRTR | CCAAGATCATCTCCAGTGCC |
| 5651qRTF | ACCCGTTGCTGCCGATGG |
| 5651qRTR | TAGACCTGGAAGAGGATGTTGTGG |
| Tub1RTF | CAACATCCAGACCGCTCTC |
| Tub1RTR | ACCGACACGCTTGAACAG |
| 446AF | GCCCAACCTTTCATCCTA |
| 446AR | TTGACCTCCACTAGCTCCAGCCAAGCCTACCTCCAGTGCCTCCTT |
| 446BF | GAATAGAGTAGATGCCGACCGCGGGTTCTCGTCCAACTCTAAACTAAC |
| 446BR | GCTGGGTAAACATCTCATT |
| 5651AF | GGGGTACCCCCTCTAAGTGGTCGTGC |
| 5651AR | CCGGAATTCCTTGGATGGAATTGTGCC |
| 5651BF | CGCGGATCCAGGGAGGCGTTATCATTTA |
| 5651BR | TAATCTAGACAGAGCCGAGCTTGTCTA |
| 446comF | GCTCTAGAGCGGAACCAGTAGTTGACGG |
| 446comR | GGGGTACCCATGACAACGCCGGAGGG |
| 5651comF | GCTCTAGAGCCCGACAAGCACAAAAGAT |
| 5651comR | CCCCCGGGGAGCGTTCGTTTAGACCC |
| 3696GFPF | CGGGATCCATGCACAGCATGGCACGC |
| 3696GFPR | CGGAATTCTGTTGAAATTACCAGCGATTC |
| 5651GFPF | CGGGATCCATGGAAGATTTTGGCAGCG |
| 5651GFPR | CCCTCGAGTCAGACACCTTGCATCATG |
