# Supplementary figures and images for: Magnaporthe oryzae CK2 Accumulates in Nuclei, Nucleoli, at Septal Pores and Forms a Large Ring Structure in Appressoria, and Is Involved in Rice Blast Pathogenesis
Source: Front Cell Infect Microbiol. 2019 Apr 17;9:113. doi: 10.3389/fcimb.2019.00113 (PMC6478894; doi:10.3389/fcimb.2019.00113)

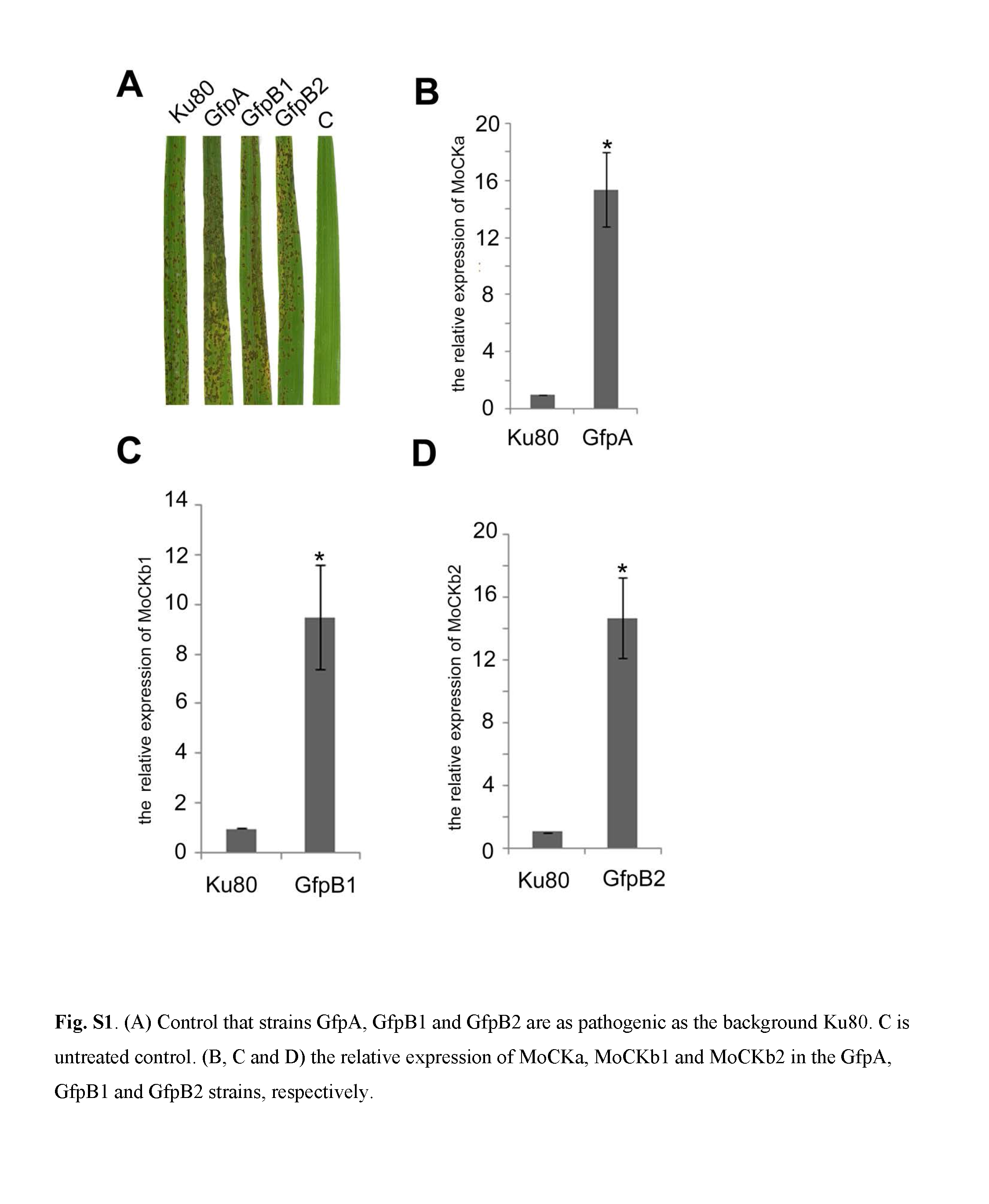

Supplement: Figure S1 — (A) Control that strains GfpA, GfpB1, and GfpB2 are as pathogenic as the background Ku80. C is untreated control. (B–D) the relative expression of MoCKa, MoCKb1, and MoCKb2 in the GfpA, GfpB1, and GfpB2 strains, respectively. [file Image_1.TIFF]

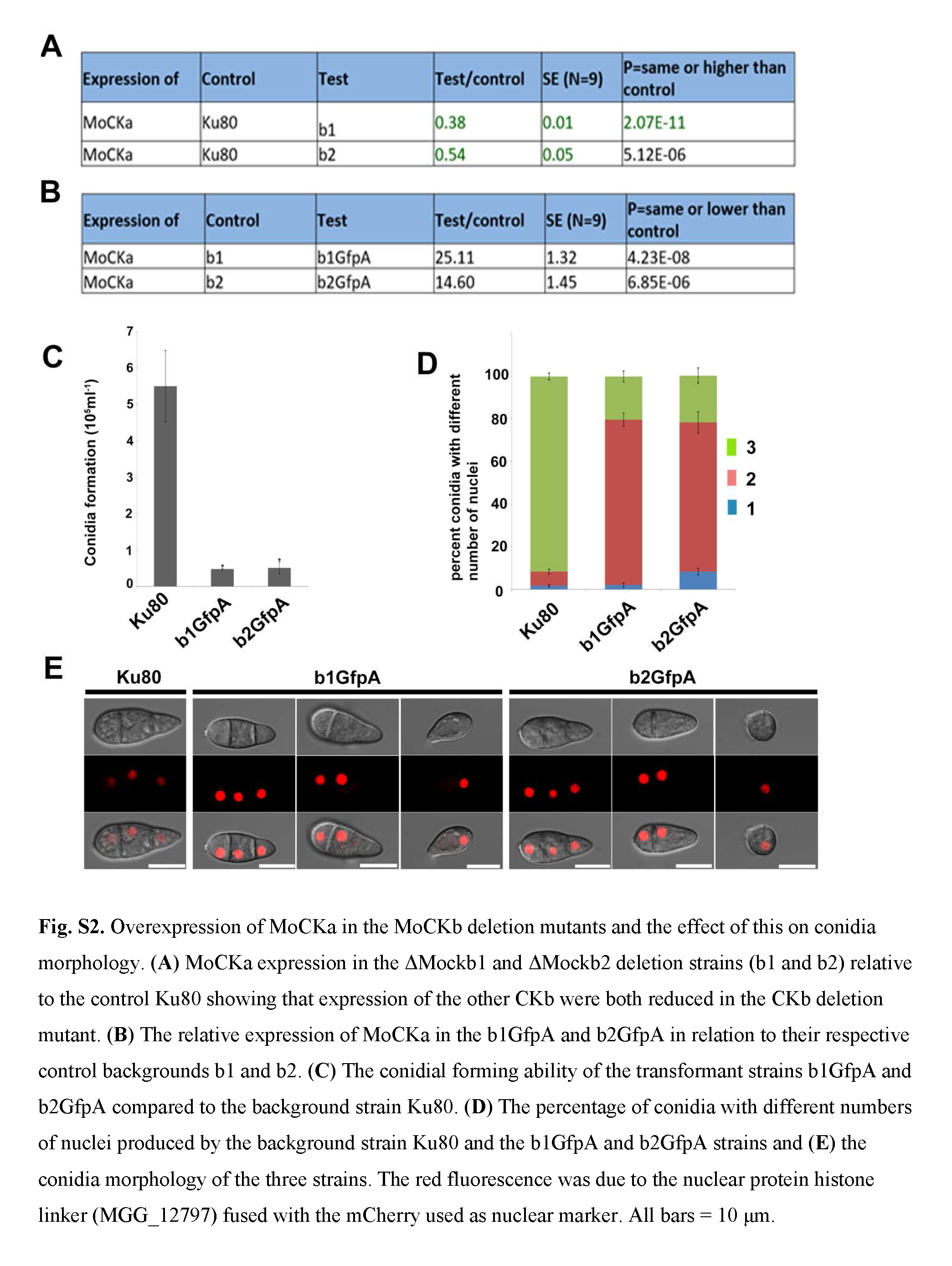

Supplement: Figure S2 — Overexpression of MoCKa in the MoCKb deletion mutants and the effect of this on conidia morphology. (A) MoCKa expression in the ΔMockb1 and ΔMockb2 deletion strains (b1 and b2) relative to the control Ku80 showing that expression of the other CKb were both reduced in the CKb deletion mutant. (B) The relative expression of MoCKa in the b1GfpA and b2GfpA in relation to their respective control backgrounds b1 and b2. (C) The conidial forming ability of the transformant strains b1GfpA and b2GfpA compared to the background strain Ku80. (D) The percentage of conidia with different numbers of nuclei produced by the background strain Ku80 and the b1GfpA and b2GfpA strains, and (E) the conidia morphology of the three strains. The red fluorescence was due to the nuclear protein histone linker (MGG_12797) fused with the mCherry used as nuclear marker. All bars = 10 μm. [file Image_2.TIFF]
